# Forty years of advances in optical biosensors—are “autonomous” biosensors in our future?

**DOI:** 10.1007/s00216-024-05338-1

**Published:** 2024-05-30

**Authors:** Frances S. Ligler, George T. Ligler

**Affiliations:** 1 Department of Biomedical Engineering, Texas A&M University, College Station, TX USA; 2 Department of Multidisciplinary Engineering, Texas A&M University, College Station, TX USA

**Keywords:** Optical biosensors, Smart tattoo, Embedded sensors, Autonomous biosensors

## Abstract

Optical biosensors have employed at least three distinct system architectures over the last 40 years, moving from “sample in-answer out” systems to completely embedding the optical biosensor into the sample to embedding the recognition module in the sample and optically interrogating the recognition module from outside of the sample. This trends article provides an overview of the evolution of these three system architectures and discusses how each architecture has been applied to solve the measurement challenges of a wide variety of applications. A fourth biosensor system architecture, that of an “autonomous” biosensor which “takes the user out of the loop” while both detecting target analytes and responding to that measurement, is currently under development for applications initially including environmental cleanup and “smart therapeutics.” As is the case in many other areas of technology, it will be profoundly interesting to observe the further development and application of elegant, simpler (optical) biosensor systems to address tomorrow’s measurement needs.

**Graphical abstract:**

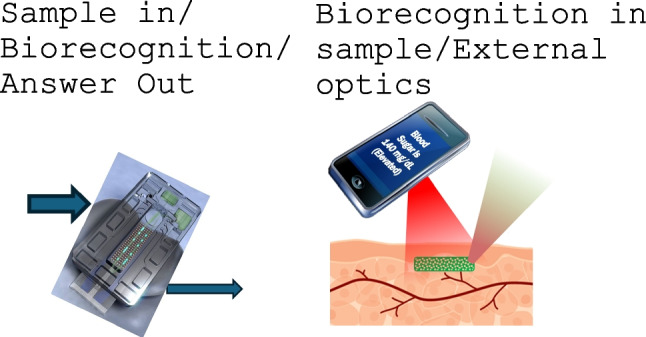

## Introduction

This trends article is dedicated to our friend and colleague Maria Moreno-Bondi [1, 2]. Maria spent a lifetime working internationally as well as in Madrid learning new chemical approaches to create practical strategies for optical sensors. Initially, she moved to Austria to join the lab of Prof. Otto Wolfbeis and contributed to his early work establishing the use of fluorescence probes in optical biosensors [1]. In the early 2000s, she expanded her travels to work with Tuan Vo-Dinh in Tennessee and Fran Ligler in Washington DC to develop multiplexed immunosensors for applications such as monitoring disease exposure and vaccination efficacy [2, 3]. While she continued to use multiplexed biosensors during the remainder of her research career, she stayed at the forefront of the development of new chemistries for optical biosensors, expanding her molecular recognition expertise from antibodies to aptamers, phage-display peptides and molecularly imprinted polymers, and the targeted applications from medical diagnostics to environmental monitoring and food safety.

## Current optical biosensor configurations

**Fig. 1:**
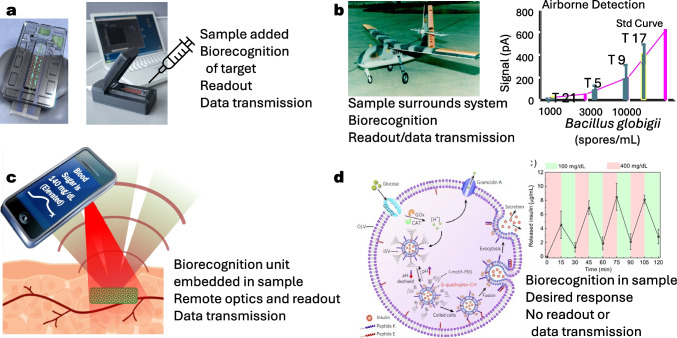
Four biosensor configurations: **a** Sample in-answer out [described in 4]. **b** System inserted into sample with answer sent to remote user [described in 5]. Data shows positive detection events during flights in four releases of the harmless *B. globigii* bacteria. **c** Recognition molecules embedded in sample and interrogated with remote optoelectronic device [described in 6]. **d** Recognition molecules and response embedded in sample with no signal transmitted to user [reprinted from 7]. Data shows the repeated ability of the liposomes to detect high glucose, release insulin, and close back up when normoglycemic conditions were detected

Optical biosensors are usually defined as portable optoelectronic devices that measure an optical signal when a biological recognition molecule binds its target. Over the last 40 years, optical biosensors have incorporated biorecognition molecules with automated fluidics and a wide variety of optical components and electronics to produce “sample in-answer out” detection systems. In this trends article, we discuss three biosensor configurations in the evolution of optical biosensors and predict a fourth potential paradigm that may put many current optical biosensor-based systems out of business. These three configurations and potential paradigm are depicted in Fig. 1.

Most of the optical biosensors currently in use today employ a similar geometry in which the sample is inserted into the biosensors and a signal is generated that can inform the user. In this configuration, biorecognition molecules are usually attached to a surface, the sample to be interrogated is passed over the surface, and the recognition molecule binds its target. Additional reagents may be included to bind to the captured target to increase specificity and sensitivity of the measurements. Portable optoelectronic devices are used to measure an optical change at the recognition site when a target is bound.

The first optical biosensors primarily used antibodies for target recognition, and one of the first major challenges was to develop methods for immobilizing proteins on optically active surfaces using chemistries that would maintain activity and prevent nonspecific adsorption/denaturation [8, 9]. Once appropriate immobilization chemistries evolved, the employment of nanobodies, lectins, oligonucleotides, carbohydrates, peptides, and molecularly imprinted polymers (MIPS) significantly increased the range of targets detectable, as well as the ability to create sensors that maintain stability under wider ranges of storage and use. Maria Moreno-Bondi was a principal leader in the integration of MIPS into optical biosensors [10, 11]. Initially, optical fibers were waveguides of choice due to the ease of controlling the excitation and emission light paths, but planar waveguides are largely preferred now because (1) they are easier and less expensive to manufacture, (2) simpler to modify, (3) appropriate for multiplexed analyses, and (4) more amenable to integration with other components [12, 13]. The measured optical change can be caused by a either a refractive index change (e.g., reflectance, surface plasmon resonance, interferometry) or a change in the mass of a surface film (e.g., ellipsometry) or can be a change in intensity at a particular wavelength (e.g., colorimetry, fluorescence) or a change in spectroscopic signature (e.g., Raman). The integration of molecular tags, such as fluorophores, phosphors, nanoparticles, and enzymic amplification constructs may increase complexity but are highly useful both in discriminating recognition events in the presence of unpredictable sample matrices and for signal amplification to increase detection sensitivity. Microfluidics are now integrated into the majority of these biosensors to automate operations such as the delivery of the sample to the biological detection molecules, washing away unbound components, and introducing signal amplification molecules. Significant advances in functionality and manufacturability as well as miniaturization of the sensors have been facilitated as new substrate geometries have been created that can house organic photonics at the same time as they channel fluids and guide light (e.g., [4]). Electronic devices measure the change and provide that information to the user either directly at the point of use or by telemetry to a remote site. Frequently, biosensors integrate technology developed for cell phone optics or communications directly into the sensor or employ entire cell phones for imaging and communications [14].

Lateral flow tests are the simplest version of an optical biosensor, usually employing antibodies as recognition molecules, nanoparticles as amplification tags, paper fluidics, room light for excitation, and visual detection. While lateral flow assays have become well known for pregnancy tests and diagnosing COVID-19 and are being employed for a broad range of other medical diagnostics, their sensitivity and capacity for multiplexed analyses is limited. Thus there is a real need for more sophisticated analytical biosensors that can provide increased sensitivity and specificity and test for multiple targets simultaneously. Low cost devices that can diagnose multiple diseases simultaneously have been under commercialization for over 20 years [15] and being continuously improved [5, 16, 17].

In addition to medical applications, portable optical biosensors using the “sample in-answer out” configuration have demonstrated value for environmental monitoring and remediation, food safety, explosive detection, biowarfare defense, and detecting drugs of abuse [18–20]. As these sensors decrease in cost and become increasingly user friendly, they will be employed in ensemble activities over broader geographic areas. Examples where point-of-use detection has been demonstrated and could be expanded to multipoint applications could include mapping the spread of pollutants in real time, assessing population health in animals as well as humans, monitoring water quality, and facilitating precision agriculture.

Though nearly all commercial optical biosensors involve putting the sample into the sensor, there are examples of a second configuration in which the entire sensor, including the molecular recognition and optoelectronics, is embedded in the sample, reminiscent of the 1966 movie Fantastic Voyage (where a miniaturized submarine was introduced into a human to repair brain damage.) In the mid-1990s, a fiber optic biosensor in a small drone demonstrated the ability to detect bacteria remotely and in midair [21]. Subsequently, a flow immunosensor in an unmanned underwater vehicle detected explosives leaking from simulated underwater mines [22]. In all of these examples, the entire analyses were performed inside the samples and the processed data transmitted remotely to the user. The advantages were that the measurements could be made repeatedly for extended periods of time, all processing was automated, and there was no requirement for the user to be exposed to the sample. Thus, this configuration is particularly well suited for continuous monitoring for toxic substances.

The introduction of entire biosensors into the human body, however, is fraught with problems. In 2004, US patent No. 6,673,596 described an optical biosensor involving gene recognition and luminescent signal generation on a chip fully implanted in the body [23]. However, the ability of a mammal to wall off a foreign body with a layer of impenetrable scar-like tissue is well known, limiting the commercial potential of this device. In addition, the introduction of hard, electronic materials usually generates toxicity. While investigators such as John Rogers [24] are developing biocompatible, biodegradable electronic materials that may address some of these issues for fully implantable biosensors, a third configuration has been demonstrated that can provide for continuous in vivo sensing. In this configuration, the recognition element is embedded in the sample and optically interrogated using optoelectronics positioned external to the sample.

The first versions of this configuration used optical fibers to carry light between the optoelectronics and the sensing element. As long ago as 1994, the Saylor group embedded a fiber optic tipped with bioluminescent bacteria in ground water to detect environmental pollutants [6]. In a similar configuration for a very different application, carbon dioxide-sensitive hydrogels located at the end of an optical fiber continuously monitor fermentation processes to optimize performance [25].

More recently, examples that completely separate the sensing element from the optoelectronics have been demonstrated for in vivo use. The first example of such a sensor configuration was a glucose sensor for diabetics embedded in a contact lens that was interrogated using an external colorimetric monitor [7]. Unfortunately, while the sensor did measure glucose, tears turned out to be unreliable for reflecting glucose concentrations in the body as a whole. Peripheral artery disease is an application where sensing a local area in vivo offers an approach to measure therapeutic efficacy and possibly prevent complications such as nonhealing ulcers and lower limb amputation. To address this problem, Michael McShane developed implantable oxygen sensors from oxygen-sensitive phosphors embedded in hydrogels. These optical sensor elements, for which he coined the term “smart tattoos” [26], are biocompatible, functional for months to years, addressable using external optics, and now produced commercially [27]. The McShane and Cote groups have continued this line of engineering, fabricating and testing implantable sensors for glucose and other targets, and creating tiny hydrogel fibers capable of multiplexed measurements [28].

## Outlook

Looking at these geometric configurations over half a century of development of optical biosensors is intriguing [29]. As an alternative to a requirement to collect a sample and add it to a biosensor, we can now add our recognition molecules to the sample and detect spectral changes using a CMOS-based imager or spectrometer. In the most advanced configuration, the detector can be both portable and remote from the recognition molecules, and direct contact with the sample is not required. This geometric paradigm suggests the potential for an entire new range of measurements for optical biosensors. For the first time, we can envision continuous, long-term measurements in living cells, three-dimensional tissues, and even intact animals. We no longer need to extract, fix, or terminate living organisms in order to perform functional measurements. Furthermore, imaging capabilities, including those based on smartphone technology, suggest that we can analyze larger areas, such as thousands of individual cells in complex arrangements, simultaneously, without reducing the data to “average values” [30].

Other exciting research advances suggest an even more game-changing fourth configuration. What if we could sense and respond without any optoelectronic device and no need to inform the user of the presence or concentration of a target analyte? Cells do this naturally. Investigators such as Gary Sayler, Shimshon Belkin, and Sylvia Daunert have been modifying bacteria for decades to luminesce when they bind heavy metals and other environmental pollutants in ground water—why not further modify the *same* strains of bacteria to simultaneously sense and remediate the pollutants? One could envision trapped bacteria that could monitor pollutant levels if needed, but would solve the problem regardless of whether an operator was present or not. Similarly, a liposomal construct is being commercialized that releases insulin in vivo only when glucose levels are high [31]. These insulin-releasing liposomes are embedded into dissolvable microneedles in a patch that is simply attached to the skin each day. No monitoring is required to achieve normal glucose levels. Hopefully, these types of advances will usher in a new generation of “autonomous” biosensors for some applications. For many if not most applications, the answer really is the most effective, lowest cost sensor irrespective of the underlying technology. Nonetheless, easier and simpler ways to solve measurement challenges require new ways of thinking, and, at least for most customers, the simpler and more automatic—requiring minimal thought or action, the better.

## References

[1] Moreno-Bondi MC, Wolfbeis OS. Oxygen optrode for use in a fiber-optic glucose biosensor. Anal Chem. 1990;52:2377–80. 10.1021/ac00220a021 2291483

[2] Moreno-Bondi MC, Alarie JP, Vo-Dinh T. Multi-analyte analysis system using an antibody-based biochip. Anal Bioanal Chem. 2003;375:230–5. 10.1007/s00216-002-1626-y 12520447

[3] Moreno-Bondi MC, Taitt CR, Shriver-Lake LC, Ligler FS. Multiplexed measurement of serum antibodies uisning an array biosensor. Biosens Bioelectron. 2006;21:1880–6. 16434176 10.1016/j.bios.2005.12.018

[4] Wojciechowski J, Shriver-Lake LC, Yamaguchi M, Fuereder E, Pieler R, Schamesberger M, Winder C, Prall H, Sonnleitner M, Ligler FS. Organic photodiodes for biosensor miniaturization. Anal Chem. 2009;81:3455–61. 19331380 10.1021/ac8027323

[5] Rosa BG, Akingbade OE, Guo X, Gonzalez-Macia L, Crone MA, Cameron LP, Freemont P, Choy K-L, Guder F, Yeatman E, Sharp DJ, Li B. Multiplexed immunosensors for point-of-care diagnostic applications. Biosens Bioelectron. 2022;203: 114050. 10.1016/j.bios.2022.114050. 35134685 10.1016/j.bios.2022.114050

[6] Heitzer A, Malachowsky K, Thonnard JE, Bienkowski PR, White DC, Sayler GS. Optical biosensor for environmental on-line monitoring of naphalene and salicylate bioavailability with an immobilized bioluminescent catabolic reporter bacterium. Applied Environ Microbiol. 1994;60:1487–94. 10.1128/aem.60.5.1487-1494.1994 PMC201507 8017932

[7] Elsherif M, Hassan MU, Yetisen AK, Butt H. Wearable contact lens biosensors for continuous glucose monitoring using smartphones. ACS Nano. 2018;12:5452–62. 29750502 10.1021/acsnano.8b00829 PMC6107296

[8] Bhatia SK, Shriver-Lake LC, Prior KJ, Georger JH, Calvert JM, Bredehorst R, Ligler FS. Use of thiol-terminal silanes and heterobifunctional crosslinkers for immobilization of antibodies on silica surfaces. Anal Biochem. 1989;178:408–13. 2546467 10.1016/0003-2697(89)90662-3

[9] Prime KL, Whitesides GM. Self-assembled organic monolayers: model systems for studying adsorption of proteins at surfaces. Science. 1991;252:1164–7. 2031186 10.1126/science.252.5009.1164

[10] Urraca JL, Marazuela MD, Merino ER, Orellana G, Moreno-Bondi MC. Molecularly imprinted polymers with a streamlined mimic for zearalenone analysis. J Chromatography A. 2006;1115:127–134. 10.1016/j.chroma.2006.03.032 16595138

[11] Urraca JL, Moreno-Bondi MC, Hall AJ, Sellergren B. Direct extraction of penicillin G and derivatives from aqueous samples using a stoichiometrically imprinted polymer. Anal Chem. 2007;79:695–701. 17222039 10.1021/ac061622r

[12] Benito-Pena E, Valdes MG, Glahn-Martinez BG, Moreno-Bondi MC. Fluorescence based fiber optic and planar waveguide biosensors. A review. Anal Chim Acta. 2016;943:17–40. 27769374 10.1016/j.aca.2016.08.049 PMC7094704

[13] Taitt CR, Anderson GP, Ligler FS. Evanescent wave fluorescence biosensors. Biosens Bioelectron. 2005;20:2470–87. 15854820 10.1016/j.bios.2004.10.026

[14] Tseng D, Mudanyali O, Oztoprak C, Isikman SO, Sencan I, Yaglidere O, Ozcan A. Lensfree microscopy on a cellphone. Lab Chip. 2010;10:1787–92. 20445943 10.1039/c003477k PMC2941438

[15] Lochhead MJ, Todorof K, Delaney M, Ives JT, Greef C, Moll K, Rowley K, Vogel K, Myatt C, Zhang X-Q, Logan C, Benson C, Reed S, Schooley RT. Rapid multiplexed immunoassay for simultaneous serodiagnosis of HIV-1 and coinfections. J Clin Microbiol. 2011;49:3584–3490. 21865431 10.1128/JCM.00970-11 PMC3187301

[16] Chen YT, Lee YC, Lai YH, Lim JC, Huang NT, Lin CT, Huang JJ. Review of integrated optical biosensors for point-of-care applications. Biosensors. 2020;10:209. 33353033 10.3390/bios10120209 PMC7766912

[17] Chaturvedi N, Goncharov A, Tripathy S, San Juan AMT, Mabbott SB, Ozcan A, Ligler FS, Cote GL. Advances in point-of-care optical biosensing for underserved populations. Trends Anal Chem. 2024;in press. 10.1016/j.trac.2024.117731.

[18] Damborsky P, Svitel J, Katrlik J. Optical biosensors. Essays Biochem. 2016;60:91–100. 27365039 10.1042/EBC20150010 PMC4986466

[19] Chadha U, Bhardwaj P, Agarwal R, Rawat P, Agarwal R, Gupta I, Panjwani M, Singh S, Ahuja C, Selvaraj SK, Banavoth M, Sonar P, Badoni B, Chakravorty A. Recent progress and growth in biosensors technology: a critical review. J Ind Eng Chem. 2022;109:21–51.

[20] Singh AK, Mittal S, Das M, Saharia A, Tiwari M. Optical biosensors: a decade in review. Alex Eng J. 2023;67:673–91.

[21] Ligler FS, Anderson GP, Davidson PT, Foch RJ, Ives JT, King KD, Page G, Stenger DA, Whelan JP. Remote sensing using an airborne biosensor. Environ Sci Technol. 1998;2:2461–6.

[22] Adams AA, Charles PT, Veitch SP, Hanson A, Deschamps JR, Kusterbeck AW. REMUS100 AUV with an integrated microfluidic system for explosives detection. Anal Bioanal Chem. 2013;405:5171–8. 23539095 10.1007/s00216-013-6853-x

[23] Sayler GS, Simpson MI, Applegate BM, Ripp SA. In vivo biosensor apparatus and method of use. US patent 6,673,596. Jan 6, 2004.

[24] Hu Z, Guo H, An D, Wu M, Kaura A, Oh H, Wang Y, Zhao M, Li S, Wnag B, Yoo D, Tran P, Ghoreshi-Haack N, Kozorovitsky Y, Huang Y, Li R, Rogers JA. Bioresorbable multilayer organic-inorganic films for bioelectronic systems. Adv Materials. 2024. 10.1002/adma.202309421. 10.1002/adma.202309421 38339983

[25] Hetzler Z, Wang Y, Krafft D, Jamalzadegan S, Overton L, Kudenov MW, Ligler FS,Wei Q. Flexible sensor patch for continuous carbon dioxide monitoring. Front Chem. 2022;10. 10.3389/fchem.2022.983523. 10.3389/fchem.2022.983523 PMC9552331 36238093

[26] Brown JQ, McShane MJ. Modeling of spherical fluorescent glucose microsensor systems: design of enzymatic smart tattoos. Biosens Bioelectron. 2006;21:1760–9. 16219457 10.1016/j.bios.2005.08.013

[27] Aranda S. Research presented at LINC suggests the Profusa LumeeR oxygen platform may improve clinical management of patients with critical limb ischemia. 2020. https://profusa.com/profusa-presented-linc/. Accessed 7 Apr 2024.

[28] McShane MM, Russell RJ, Pishko MV, Cote GL. Glucose monitoring using implanted fluorescent microspheres. IEEE Eng Med Biol Magazine. 2000;19:36–45. 10.1109/51.887244 11103704

[29] Ligler FS, Gooding JJ. Lighting up biosensors—now and the decade to come. Anal Chem. 2019;91:8732–8. 31276374 10.1021/acs.analchem.9b00793

[30] Ozcan A, Demirci U. Ultra wide-field lens-free monitoring of cells on-chip. Lab Chip. 2008;8:98–106. 18094767 10.1039/b713695a

[31] Yu J, Zhang Y, Ye Y, DiSanto R, Sun W, Ranson D, Ligler FS, Buse JB, Gu Z. Microneedle-array patches loaded with hypoxia-sensitive vesicles provide fast glucose-responsive insulin delivery. Proc Nat Acad Sci USA. 2015;112:8260–5. 26100900 10.1073/pnas.1505405112 PMC4500284

